# Dual Specificity Phosphatase 6 Protects Neural Stem Cells from β-Amyloid-Induced Cytotoxicity through ERK1/2 Inactivation

**DOI:** 10.3390/biom8040181

**Published:** 2018-12-19

**Authors:** Wang Liao, Yuqiu Zheng, Wenli Fang, Shaowei Liao, Ying Xiong, Yi Li, Songhua Xiao, Xingcai Zhang, Jun Liu

**Affiliations:** 1 Department of Psychiatry, McLean Hospital, Harvard Medical School, Belmont, MA 02478, USA; docliaowang@foxmail.com; 2 Guangdong Province Key Laboratory of Brain Function and Disease, Zhongshan School of Medicine, Sun Yat-sen University, Guangzhou 510120, China; zhengyq6@mail2.sysu.edu.cn (Y.Z.); fangwl@mail2.sysu.edu.cn (W.F.); liaoshaowei2015@163.com (S.L.); xyzjy09154013@163.com (Y.X.); liyi_lynn@126.com (Y.L.); xiaosh2017@163.com (S.X.); 3 John A Paulson School of Engineering and Applied Science, Harvard University, Cambridge, MA 02138, USA

**Keywords:** Alzheimer’s disease, DUSP6, Aβ, ER stress, mitochondrial, ERK, oxidative stress

## Abstract

Alzheimer’s disease (AD) is a devastating neurodegenerative disease with limited treatment options and no cure. Beta-amyloid (Aβ) is a hallmark of AD that has potent neurotoxicity in neural stem cells (NSCs). Dual specificity phosphatase 6 (DUSP6) is a member of the mitogen-activated protein kinases (MAPKs), which is involved in regulating various physiological and pathological processes. Whether DUSP6 has a protective effect on Aβ-induced NSC injury remains to be explored. C17.2 neural stem cells were transfected with DUSP6-overexpressed plasmid. NSCs with or without DUSP6 overexpression were administrated with Aβ25–35 at various concentrations (i.e., 0, 2.5, 5 μM). DUSP6 expression after Aβ treatment was detected by Real-Time Polymerase Chain Reaction (RT-PCR) and Western blot and cell vitality was examined by the CCK8 assay. The oxidative stress (intracellular reactive oxygen species (ROS) and malondialdehyde (MDA)), endoplasmic reticulum stress (ER calcium level) and mitochondrial dysfunction (cytochrome c homeostasis) were tested. The expression of *p*-ERK1/2 and ERK1/2 were assayed by Western blot. Our results showed that Aβ decreased the expression of DUSP6 in a dose-dependent manner. The overexpression of DUSP6 increased the cell vitality of NSCs after Aβ treatment. Oxidative stress, ER stress, and mitochondrial dysfunction induced by Aβ could be restored by DUSP6 overexpression. Additionally, the Aβ-induced ERK1/2 activation was reversed. In summary, DUSP6 might have a neuroprotective effect on Aβ-induced cytotoxicity, probably via ERK1/2 activation.

## 1. Introduction

Alzheimer’s disease (AD) is the most prevalent cause of dementia and affects 1 in 10 people over 65 years of age [1]. Currently, there are no known drugs that are able to stop the progressive cognitive decline associated with AD. Additionally, no new drugs have been approved by the Food and Drug Administration (FDA) since 2003 [2]. However, neural stem cell (NSC) therapy brings new hope to AD treatment. Adult neurogenesis is a process that generates new neural cells, which could be a promising therapeutic approach for neurodegenerative disease [3]. However, NSCs are susceptible to physiological or pathological microenvironment change [4].

One of the hallmarks of AD is the extracellular accumulation of beta-amyloid (Aβ), which has potent neurotoxicity on the central nervous system [5]. Therefore, it is crucial to prevent NSCs from this neurotoxicity. Multiple studies have shown ways to protect NSCs against Aβ, like mitochondrial protein (sirtuin3) [6] and natural herbs (incensole acetate) [7]. Our previous study demonstrated that dual specificity phosphatase 6 (DUSP6), a member of the dual specificity protein phosphatase subfamily, had a protective property against glutamate-induced cytotoxicity in mouse hippocampal neurons [8]. However, there is no evidence concerning the effects of DUSP6 in Aβ-induced cytotoxicity. Interestingly, the protein level of DUSP6 was found to be decreased in AD brain lysates and DUSP6 knockdown increased the level of phospho-ERK to promote high levels of tau phosphorylation [9,10]. Prior results indicate that DUSP6 could be a treatment target for AD.

The occurrence of AD is a multifactorial pathological process with many risk factors involved [11]. Aβ aggregation induces oxidative stress, and increased reactive oxygen species (ROS) facilitate Aβ generation, which consequently exacerbates cell damage [12]. Mitochondrial dysfunction plays a vital role in the pathological process of AD due to the high energy demand of the central nervous system [13]. Additionally, AD is associated with endoplasmic reticulum stress, which is thought to trigger cell death under Aβ exposure [14]. In the present study, we investigate the role of DUSP6 in Aβ-induced cytotoxicity in NSCs and the underlying molecular mechanisms involving oxidative stress, mitochondrial dysfunction, and reticulum stress.

## 2. Results

### 2.1. β-amyloid Treatment Reduced DUSP6 Expression at Different Concentrations

**Figure 1:**
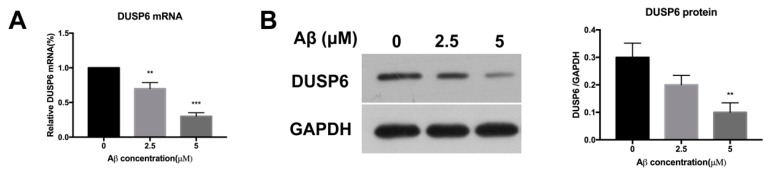
Beta amyloid (Aβ)25–35 treatment reduced dual specificity phosphatase 6 (DUSP6) expression at different concentrations. (**A**) After treated with Aβ25–35 (0, 2.5, 5 μM) for 24 h, DUSP6 mRNA in neural stem cells (NSCs) was measured by RT-PCR; (**B**) DUSP6 protein was collected for Western blot assay. **: *p* < 0.01, ***: *p* < 0.001 vs. 0 μM. *n* = 3. GAPDH: Glyceraldehyde-3-Phosphate Dehydrogenase.

To explore the effect of Aβ-induced cytotoxicity in NSCs, DUSP6 mRNA was measured by RT-PCR after being treated with Aβ25–35 (0, 2.5, 5 μM) for 24 h. As shown in Figure 1A, DUSP6 mRNA was significantly reduced after Aβ25–35 treatment at concentrations of both 2.5 and 5 μM (*p* < 0.01, *n* = 3). Consistently, DUSP6 protein expression detected by Western blot assay showed a decrease in the Aβ25–35-treated group (5 μM) (*p* < 0.01, *n* = 3) (Figure 1B), indicating that DUSP6 might play an important role in Aβ-induced cytotoxicity in NSCs.

### 2.2. DUSP6 Prevented the beta-amyloid25–35-Induced Decrease in Neural Stem Cell Viability

**Figure 2:**
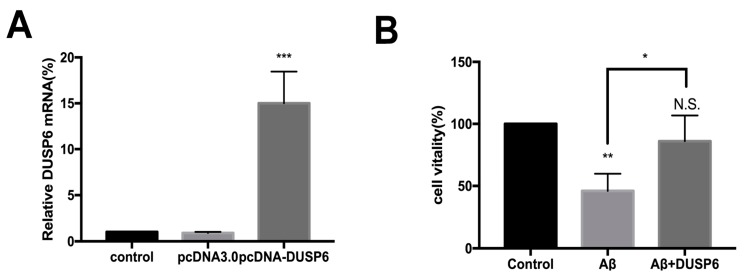
DUSP6 prevented the Aβ-triggered decrease in NSC viability. (**A**) DUSP6 mRNA was overexpressed after it was transfected with DUSP6-overexpressed plasmid pcDNA-DUSP6. pcDNA3.0 empty vector transfection showed no effect on DUSP6 expression in NSCs; (**B**) NSCs were either pre-transfected with DUSP6 overexpression plasmid or not transfected. Both groups were treated with 5 μM Aβ25–35 for 24 h. Cell viability assessed by CCK8 assay showed no significant change in Aβ-treated NSC vitality after DUSP6 transfection. N.S.: *p* > 0.05, **: *p* < 0.01, ***: *p* < 0.001 vs. control. *: *p* < 0.05 vs. Aβ-treated group. *n* = 3. N.S.: Not significant.

To further determine the role of DUSP6 in Aβ-induced cytotoxicity, DUSP6 overexpression plasmid was constructed and transfected into NSCs. Figure 2A shows that DUSP6 mRNA was overexpressed after being transfected with DUSP6 overexpression plasmid pcDNA-DUSP6 for 24 h (*p* < 0.001, *n* = 3). In contrast, pcDNA3.0 empty vector transfection showed no effect on DUSP6 expression in NSCs (*p* > 0.05).

The non-transfected NSCs and DUSP6 plasmid-transfected NSCs were exposed to 5 μM Aβ25–35 for 24 h. Non-transfected cells were regarded as a control, and cell viability was considered to be 100%. Cell viability assessed by CCK8 assay showed a significant decrease in the Aβ-treated group (*p* < 0.01, *n* = 3) (Figure 2B). However, there was no significant change in the vitality of Aβ-treated NSCs after DUSP6 transfection (*p* > 0.05). Hence, our results indicate that DUSP6 could potentially prevent the effect of Aβ25–35 on the viability of NSCs.

### 2.3. DUSP6 Restored Reduced Intracellular Reactive Oxigen Species and Malondialdehyde Level in Neural Stem Cells After beta amyloid25–35 Treatment

**Figure 3:**
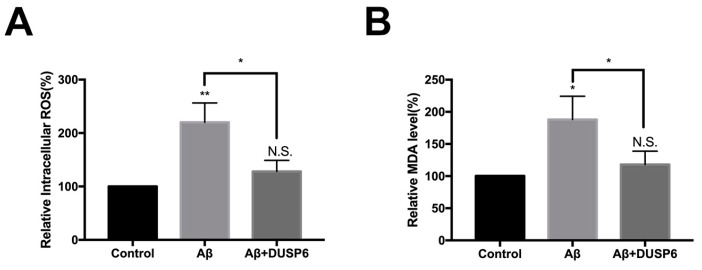
DUSP6 reduced Aβ-induced oxidative stress in NSCs. NSCs were either transfected with DUSP6 overexpression plasmid or not transfected. Both groups were treated with 5 μM Aβ25–35 for 24 h and then admitted to intracellular reactive oxygen species (ROS) (**A**) and lipid peroxidation marker malondialdehyde (MDA) assay (**B**). The values were normalized to the control group. N.S.: *p* > 0.05, *: *p* < 0.05, **: *p* < 0.01 vs. control. *: *p* < 0.05 vs. Aβ25–35 treated group. *n* = 3.

Some prior literature reported that oxidative stress plays a vital role in AD [15]. In the present study, we showed that both intracellular ROS and lipid peroxidation marker malondialdehyde (MDA) levels were increased after Aβ25–35 treatment (Figure 3A, B) (*p* < 0.05, *n* = 3). Notably, there was no difference between levels of intracellular ROS and MDA in the DUSP6-overexpressed group compared with that in the control group (*p* > 0.05). We observed a distinction between the Aβ25–35-exposed group and the DUSP6-transfected group (*p* < 0.05, *n* = 3), suggesting that the effect of Aβ on intracellular ROS and MDA levels could be abrogated by DUSP6.

### 2.4. DUSP6 Reversed Aβ-Induced Effect on ER Calcium and Mitochondrial Cytochrome c Homeostasis in Neural Stem Cells

**Figure 4:**
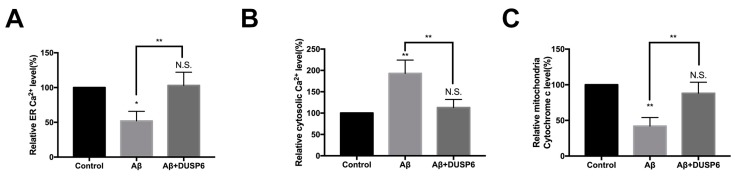
DUSP6 reversed Aβ-induced effect on ER Ca^2+^ level and mitochondrial cytochrome c homeostasis in NSCs. NSCs were either pre-transfected with DUSP6 overexpression plasmid or not transfected. Both groups were treated with 5 μM Aβ25–35 for 24 h. (**A**) Endoplasmic reticulum (ER) calcium was detected by fluorescent probe Fura-2/AM; (**B**) Cytosolic Ca^2+^ content in NSCs was measured with Indo-1/AM. (**C**) An enzyme-linked immunosorbent assay (ELISA) kit was adopted for the assay of cytochrome c in NSCs’ mitochondria. N.S.: *p* > 0.05, *: *p* < 0.05, **: *p* < 0.01 vs. control. **: *p* < 0.01 vs. Aβ-treated group. *n* = 3.

Since Ca^2+^ can be released from the endoplasmic reticulum (ER) during stress [16], we assessed the content both in the ER and cytosol to determine the effect of Aβ25–35 on NSCs after DUSP6 overexpression. As shown in Figure 4A,B, the levels of Ca^2+^ decreased significantly in the ER (*p* < 0.05, *n* = 3) while they increased in the cytosol after Aβ treatment (*p* < 0.01, *n* = 3). Notably, the effect of Aβ on ER stress and cytosol Ca^2+^ homeostasis was reversed by DUSP6 (*p* > 0.05).

Alteration in mitochondria-released cytochrome c is an indicator of cell death [17]. Hence, in this study, ELISA (Enzyme-Linked Immunosorbent Assay) was performed to detect cytochrome c in NSCs. Our results showed that while cytochrome c was reduced after Aβ25–35 treatment (*p* < 0.01, *n* = 3) (Figure 4C), this effect could be reversed by DUSP6 (*p* > 0.05, *n* = 3).

### 2.5. DUSP6 Regulated ERK1/2 Activation in Aβ25–35-Exposed Neural Stem Cells

**Figure 5:**
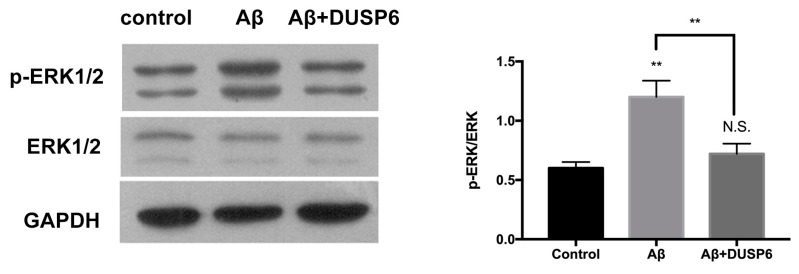
The effect of Aβ25–35 and DUSP6 overexpression on ERK1/2 activation in NSCs. NSCs were transfected with the DUSP6 overexpression plasmid. After transfection for 24 h, each group of cells was treated with Aβ25–35 for 24 h and harvested for Western blotting. N.S.: *p* > 0.05, **: *p* < 0.01 vs. control. **: *p* < 0.01 vs. the Aβ-treated group. *n* = 3.

Previous studies have reported that DUSP6 inactivated ERK1/2 [18]. Therefore, we assessed the expression of ERK1/2 activation by Western blot analysis after Aβ25–35 exposure with or without DUSP6 overexpression. We found that the ratio of *p*-ERK/ERK was significantly up-regulated in Aβ25–35-exposed NSCs when compared with the control group. After DUSP6 transfection, *p*-ERK/ERK was significantly down-regulated when compared with that in Aβ25–35 treated group (*p* < 0.01, *n* = 3) (Figure 5). There was no significant change between the control and DUSP6-overexpressed groups (*p* > 0.05). This suggests that DUSP6 might protect NSCs from Aβ25–35 induced cytotoxicity by modulating ERK1/2 signaling.

## 3. Discussion

Currently, there are two main types of Food and Drug Administration (FDA)-approved drugs available for the treatment of AD: cholinesterase inhibitors and N-Methyl-D-aspartate receptor (NMDAR) antagonists [19]. However, these drugs are not able to halt the progression of AD [20]. Immunotherapy targeting Aβ or senile plaques have also been unsuccessful in reversing the cognitive function of AD patients. Instead of cleaning Aβ, generating neural cells may be a new approach to AD treatment [21].

There are several types of stem cells being considered as a therapeutic strategy for AD, such as mesenchymal stem cells (MSCs), NSCs, induced pluripotent stem cells (iPSCs), etc. [22]. Among them, autogenous neural stem cells are most useful, given their ethical, moral, and safety advantages [21]. Adult neurogenesis is a process by which new neurons are generated throughout life [23]. The adult-born neurons affect brain function; thus, their dysfunction correlates with AD progression [24]. However, NSCs are vulnerable to microenvironmental changes in the brain. Our previous research demonstrated that magnesium elevation promotes neural differentiation and suppresses glial differentiation in neural stem cells [25]. Additionally, NSCs are sensitive to neurotoxicity [7].

In the present study, we showed that Aβ25–35 decreased the mRNA and protein expression of DUSP6 in C17.2 NSCs (Figure 1). Overexpression of DUSP6 restored cell vitality (Figure 2), oxidative stress (Figure 3), ER stress, and mitochondrial function (Figure 4), which were triggered by Aβ25–35 exposure. The Aβ25–35-induced ERK1/2 activation was also reversed by DUSP6 transfection (Figure 5). Collectively, DUSP6 may have a neuroprotective effect on Aβ25–35-induced cytotoxicity in NSCs via ERK inactivation.

AD is characterized by two main pathological markers: senile plaques and neurofibrillary tangles [26]. The senile plaques are formed of Aβ, which is a protein with potent neurotoxicity to neural cells, especially NSCs [27]. Among the different kinds of Aβ generated during AD pathogenesis, Aβ1–42 is the most toxic, while Aβ1–40 has the largest amount [20]. Aβ25–35, a synthetic peptide based on Aβ1–40 and Aβ1–42, was reported to have the physical and biological properties of Aβ without the need of aging before usage [28]. Our previous research demonstrated that Aβ25–35 was capable of inducing autophagy in HT22 hippocampus cells, which involved the PI3K/AKT/mTOR/p70S6K pathway [29]. Hence, Aβ25–35 was used in this study even though Aβ1–42 might be more commonly used.

Recently, Aβ1–42 was reported to affect the fate determination of NSCs [30]. It is assumed that Aβ drives neural stem proliferation and neural differentiation of human NSCs at low concentrations but is neurotoxic at high concentrations (i.e., 1 μM) [31]. The mechanism could be related to the increased GSK3β, induced by Aβ1–42. The Aβ25–35 concentrations used in the present study were 2.5 μM and 5 μM, which are much higher than 1 μM. This might be why cytotoxicity was observed.

It is very important to not only understand the mechanism of Aβ cytotoxicity but to find ways to protect NSCs from such cytotoxicity. According to previous literature, natural herbs such as incensole acetate have a neuroprotective effect on NSCs [7]. However, it would be more impactful to have autogenous biomolecules that can protect NSCs against Aβ.

DUSP6 (MKP3, or Pyst1), a member of the mitogen-activated protein kinases (MAPKs), is involved in regulating various physiological and pathological processes [32]. Prior studies have shown the role of DUSP6 in cell growth, inflammation, proliferation, and stress responses [32]. In this study, we demonstrated that DUSP6 protected HT22 hippocampus cells and primary neurons from glutamate-induced cytotoxicity [8]. However, it is still uncertain as to whether DUSP6 has a protective effect on Aβ-induced NSCs.

DUSP6 is tightly regulated by stress at both transcriptional and post-transcriptional levels. Previous observations indicate that Aβ increases ERK phosphorylation [33], inducing CDK5 activation [34], which in turn causes neurodegeneration [35]. Furthermore, it has been shown that DUSP6 knockdown increases tau phosphorylation and decreases cell viability via ERK phosphorylation [9]. In fact, DUSP6 seems to be one of the downstream targets of miR-125b, which is increased in AD and the overexpression impairs learning and memory formation [9]. Of note, DUSP6 is down-regulated in post-mortem samples of AD and can directly dephosphorylate and inactivate ERK [36,37]. Moreover, the pattern of DUSP6 expression in vivo indicates a strong expression in CA1, which is one of the first areas affected in AD [38].

There are other dual-specificity phosphatases that have been reported to be involved in AD. For example, DUSP26 has been reported to stimulate Aβ42 generation by promoting amyloid precursor protein axonal transport [39]. Sanchez-Mut et al. demonstrated that DUSP22 was reduced in AD patient sand mediated tau phosphorylation and CREB activation [40].

In the present study, we found that DUSP6 had a protective effect on Aβ-induced NSC injury, including oxidative stress, ER stress, and mitochondrial dysfunction. In this study, we examined ERK1/2 activation because DUSP6 has been reported to interact specifically with ERK1/2 at dual threonine and tyrosine residues [18]. The threonine-x-tyrosine TEY motifs of these residues lead to inactivation of ERK1/2, which has been demonstrated in both yeast and human cells [18].

The mechanism of interaction between DUSP6 and ERK1/2 is still unclear. Previous studies have suggested that DUSP6 overexpression de-phosphorylated ERK1/2 by blocking the MEK1-mediated GAL4-ELK1 activation [32]. DUSP6 was reported to interact with an apoptosis and autophagy-related protein BAG3. BAG3 encodes a multifunctional protein containing a BAG domain, which binds to heat shock protein (Hsp) 70. This might explain the protective effect of DUSP6 on mitochondrial function [41]. One of the main downstream targets of ERK signaling is E twenty-six (Ets) 2. Ets2 is a transcription factor that can bind to the DUSP6 promoter, which causes the up-regulation of DUSP6 [42]. An experiment on Ets2 transgenic mice showed that Ets2 also interacted with p53 promoter region, which is vital in the process of apoptosis [43]. This may be an explanation for the role of DUSP6 in Aβ-induced apoptosis. The mechanism of how Aβ interacts with ERK1/2 also remains to be explored. One possible way that Aβ interacts with ERK1/2 would be through receptors on the cell surface. It has been demonstrated that Aβ1–42 bound to the formyl peptide receptor (FPR) on the cell membrane, enhancing the G protein-coupled receptor kinase 2 (GRK2) expression and leading to the phosphorylation of ERK1/2 at early stages [44].

In this study, we did not use specific ERK activators to mimic the Aβ effect. Additionally, it would be beneficial if DUSP6 microRNA could be adopted to downregulate DUSP6 and further explore the mechanism. We are planning to construct a DUSP6-deficient mouse model to hybridize a APP/PS1 mouse in order to further explore its effect.

In this study, we showed that DUSP6 protected NSCs against Aβ25–35-induced neurotoxicity, probably via ERK1/2 inactivation.

## 4. Materials and Methods

### 4.1. Cell Culture and Treatment

The C17.2 neural stem cell line has been widely used as an in vitro model for neurodegenerative diseases because of its capacity for self-renewal and differentiation [45]. In this study, the murine C17.2 cell line was adopted as a replacement. A C17.2 cell line (Sigma-Aldrich, St. Louis, MO, USA) was cultured in high-glucose Dulbecco’s modified Eagle’s medium (DMEM) supplemented with 10% (*v*/*v*) fetal bovine serum, 5% (*v*/*v*) horse serum, 1% glutamine, 100 ng/mL streptomycin, and 100 units/mL penicillin [46]. All reagents were purchased from Gibco Thermo Fisher Scientific Inc. (Waltham, MA, USA).

The day before Aβ25–35 treatment, cells were seeded onto a 35-mm dish at a density of 2 × 10^−5^ cells per well. Different concentrations of Aβ25–35 (2.5 and 5 μM) were added to the NSCs for 24 h.

### 4.2. CCK-8 Assay for Cell Viability

The effects of Aβ25–35 on NSCs viability were detected by CCK-8 assay (Dojin Laboratories, Kumamoto, Kyushu, Japan). according to the method we previously published [47]. Briefly, NSCs were cultured on a 96-well plate at a density of 1 × 10^4^/well for 24 h and administrated with Aβ25–35 for another 24 h. Then, the absorbance values were measured at 450 nm by a multifunctional microplate reader (SpectraMax M5, Sunnyvale, CA, USA) after being incubated at 37 °C for 2 h.

### 4.3. Cell Transfection

One day before transfection, NSCs were seeded onto a 35-mm dish at a density of 2 × 10^5^ cells per well. DUSP6 (NM_001946) overexpression plasmid was constructed and transfected as we described previously [8]. Primers used by us were as follows: 5′-cgcggatccgccaccATGATAGATACGCTCAGACCCGTGC-3′ and 5′-ccgctcgagTCACGTAGACTGCAGGGAGTCCACC-3′. Plasmids were transfected using Lipofectamine 2000 according to the manufacturer’s instructions (Invitrogen, Carlsbad, CA, USA).

### 4.4. Quantitative Real-Time PCR (RT-PCR)

NSCs were seeded onto six-well plates and received the indicated treatment. After Aβ25–35 treatment for 24 h, total RNA was extracted using Trizol reagent (Invitrogen, Carlsbad, CA, USA) according to the manufacturer’s instructions. The primer sequences for DUSP6 and Glyceraldehyde-3-Phosphate Dehydrogenase (GAPDH) were as follows: DUSP6-F: 5′-GAGGGTAGCATAGGAATAGG-3′, DUSP6-R: 5′-TCTCTTTGGCTCCACTATAC-3′, GAPDH-F: 5′-GGCCTCCAAGGAGTAAGAAA-3′, and GAPDH-R: 5′GCCCCTCCTGTTATTATGG-3′. Reverse transcription and RT-PCR were performed in accordance with the protocol recommended by the manufacturers of SYBR Green qPCR SuperMix (Invitrogen). The relative expression of mRNA was assessed by the comparative 2^−∆∆CT^ method. GAPDH was used as an internal standard.

### 4.5. Measurement of Oxidative Stress

Intracellular reactive oxygen species was measured using fluorescent probe 2,7-dichlorofluorescein diacetate (DCFH-DA) [48]. Another indicator of oxidative stress, MDA, was detected with commercial kits as described previously [49].

### 4.6. Detection of Ca^2+^ Levels

Ca^2+^ levels were measured by Ca^2+^ imaging using the fluorescent probe Fura-2/AM (Thermo Fisher Scientific Inc.) as a routine procedure. Briefly, cells were washed twice and incubated with Fura-2/AM (10 μM) for 30 min. The fluorescence was measured using a microplate reader with an excitation filter of 340 nm and an emission filter of 380 nm.

### 4.7. Enzyme-Linked Immunosorbent Assay (ELISA)

Cytochrome c of the mitochondria was measured using the cytochrome c ELISA kit (Thermo Fisher Scientific Inc.) as described previously. In brief, after being incubated on ice for 1 h, cell lysates of NSCs were homogenized in a buffer and centrifuged for 5 min (700 g). Then, mitochondrial fraction was collected from supernatants after being re-centrifuged for another 1 h at 8500 ×*g*. Lastly, the absorbance of the samples was detected with a multifunctional microplate reader at 450 nm (SpectraMax M5, Sunnyvale, CA, USA).

### 4.8. Western Blot Analysis

Western blotting and semi-quantitative analyses were performed following previously described procedures [25]. In brief, proteins of NSCs were extracted with lysis buffer for 30 min, followed by centrifugation at 14,000 rpm for 15 min at 4 °C to obtain the supernatant for Western blot analysis. Primary antibodies and dilution rates used are as follows: *p*-ERK1/2 (1:1000, Cell Signaling Technology, Danvers, MA, USA), ERK1/2 (1:1000, Cell Signaling Technology (Danvers, MA, USA)), DUSP6 (1:500, Abcam, Cambridge, MA, USA). Secondary antibodies were purchased from Cell Signaling Technology Inc. Horseradish peroxidase-conjugated secondary antibodies were used, and the bands were fixed and visualized by an ECL advanced kit. GAPDH was utilized as an internal control for protein loading and transfer efficiency. Western blot assay results reported here are representative of at least three experiments. The quantification of protein expression was analyzed by Image J (National Institutes of Health, Bethesda, MD, USA).

### 4.9. Statistical Analysis

SPSS 16.0 for Windows (SPSS Inc., Chicago, IL, USA) was used to carry out the statistical analyses. Other statistical tests were conducted using one-way analysis of variance (ANOVA) and Student’s *t*-test for comparisons between groups. The data were expressed as the mean ± SE, and differences were considered statistically significant at *p* < 0.05.

## References

[1] Cavedo E., Chiesa P.A., Houot M., Ferretti M.T., Grothe M.J., Teipel S.J., Lista S., Habert M.O., Potier M.C., Dubois B. (2018). Sex differences in functional and molecular neuroimaging biomarkers of Alzheimer’s disease in cognitively normal older adults with subjective memory complaints. Alzheimer’s Dement. J. Alzheimer’s Assoc..

[2] Cummings J., Lee G., Mortsdorf T., Ritter A., Zhong K. (2017). Alzheimer’s disease drug development pipeline: 2017. Alzheimer’s Dement..

[3] Amemori T., Jendelova P., Ruzicka J., Urdzikova L.M., Sykova E. (2015). Alzheimer’s disease: Mechanism and approach to cell therapy. Int. J. Mol. Sci..

[4] Choi S.H., Bylykbashi E., Chatila Z.K., Lee S.W., Pulli B., Clemenson G.D., Kim E., Rompala A., Oram M.K., Asselin C. (2018). Combined adult neurogenesis and BDNF mimic exercise effects on cognition in an Alzheimer’s mouse model. Science.

[5] Tournier B.B., Tsartsalis S., Rigaud D., Fossey C., Cailly T., Fabis F., Pham T., Gregoire M.C., Kovari E., Moulin-Sallanon M. (2018). TSPO and amyloid deposits in sub-regions of the hippocampus in the 3xTgAD mouse model of Alzheimer’s disease. Neurobiol. Disease.

[6] Voit R., Seiler J., Grummt I. (2015). Cooperative action of Cdk1/cyclin B and SIRT1 is required for mitotic repression of rRNA synthesis. PLoS Geneti..

[7] El-Magd M.A., Khalifa S.F., Fa A.A., Badawy A.A., El-Shetry E.S., Dawood L.M., Alruwaili M.M., Alrawaili H.A., Risha E.F., El-Taweel F.M. (2018). Incensole acetate prevents β-amyloid-induced neurotoxicity in human olfactory bulb neural stem cells. Biomed. Pharmacother..

[8] Huang X., Liao W., Huang Y., Jiang M., Chen J., Wang M., Lin H., Guan S., Liu J. (2017). Neuroprotective effect of dual specificity phosphatase 6 against glutamate-induced cytotoxicity in mouse hippocampal neurons. Biomed. Pharmacother..

[9] Banzhaf-Strathmann J., Benito E., May S., Arzberger T., Tahirovic S., Kretzschmar H., Fischer A., Edbauer D. (2014). MicroRNA-125b induces tau hyperphosphorylation and cognitive deficits in Alzheimer’s disease. EMBO J..

[10] Bhore N., Wang B.J., Chen Y.W., Liao Y.F. (2017). Critical roles of dual-specificity phosphatases in neuronal proteostasis and neurological diseases. Int. J. Mol. Sci..

[11] Takeda S. (2018). Progression of Alzheimer’s disease, tau propagation, and its modifiable risk factors. Neurosci. Res..

[12] Manoharan S., Guillemin G.J., Abiramasundari R.S., Essa M.M., Akbar M., Akbar M.D. (2016). The Role of reactive oxygen species in the pathogenesis of Alzheimer’s Disease, Parkinson’s Disease, and Huntington’s Disease: A Mini Review. Oxid. Med. Cell. Longev..

[13] Van Giau V., An S.S.A., Hulme J.P. (2018). Mitochondrial therapeutic interventions in Alzheimer’s disease. J. Neurol. Sci..

[14] Hashimoto S., Saido T.C. (2018). Critical review: Involvement of endoplasmic reticulum stress in the Aetiology of Alzheimer’s disease. Open Biol..

[15] Jiang T., Sun Q., Chen S. (2016). Oxidative stress: A major pathogenesis and potential therapeutic target of antioxidative agents in Parkinson’s disease and Alzheimer’s disease. Prog. Neurobiol..

[16] Zeeshan H.M., Lee G.H., Kim H.R., Chae H.J. (2016). Endoplasmic reticulum stress and associated ROS. Int. J. Mol. Sci..

[17] Li J., Wang Y., Du L., Xu C., Cao J., Wang Q., Liu Q., Fan F. (2014). Radiation-induced cytochrome c release and the neuroprotective effects of the pan-caspase inhibitor z-VAD-fmk in the hypoglossal nucleus. Exp. Ther. Med..

[18] Arkell R.S., Dickinson R.J., Squires M., Hayat S., Keyse S.M., Cook S.J. (2008). DUSP6/MKP-3 inactivates ERK1/2 but fails to bind and inactivate ERK5. Cell. Signal..

[19] Alzheimer’s Association (2016). 2016 Alzheimer’s disease facts and figures. Alzheimer’s Dement. J. Alzheimer’s Assoc..

[20] Morris G.P., Clark I.A., Vissel B. (2018). Questions concerning the role of amyloid-β in the definition, aetiology and diagnosis of Alzheimer’s disease. Acta Neuropathol..

[21] Sun B.L., Li W.W., Zhu C., Jin W.S., Zeng F., Liu Y.H., Bu X.L., Zhu J., Yao X.Q., Wang Y.J. (2018). Clinical research on Alzheimer’s Disease: Progress and perspectives. Neurosci. Bull..

[22] Sugaya K., Vaidya M. (2018). Stem cell therapies for neurodegenerative diseases. Adv. Exp. Med. Biol..

[23] Berry B.J., Smith A.S.T., Young J.E., Mack D.L. (2018). Advances and current challenges associated with the use of human induced pluripotent stem cells in modeling neurodegenerative disease. Cells Tissues Organs.

[24] Miyashita T., Oda Y., Horiuchi J., Yin J.C., Morimoto T., Saitoe M. (2012). Mg^2+^ block of Drosophila NMDA receptors is required for long-term memory formation and CREB-dependent gene expression. Neuron.

[25] Liao W., Jiang M., Li M., Jin C., Xiao S., Fan S., Fang W., Zheng Y., Liu J. (2017). Magnesium Elevation Promotes Neuronal Differentiation While Suppressing Glial Differentiation of Primary Cultured Adult Mouse Neural Progenitor Cells through ERK/CREB Activation. Front. Neurosci..

[26] Hane F.T., Lee B.Y., Leonenko Z. (2017). Recent progress in Alzheimer’s disease research, Part 1: Pathology. J. Alzheimers Dis..

[27] Lin C.H., Nicol C.J.B., Cheng Y.C., Chen S.J., Yen C.H., Huang R.N., Chiang M.C. (2018). Rosiglitazone rescues human neural stem cells from amyloid-β induced ER stress via PPARgamma dependent signaling. Exp. Cell Res..

[28] Song H., Huang L.P., Li Y., Liu C., Wang S., Meng W., Wei S., Liu X.P., Gong Y., Yao L.H. (2018). Neuroprotective effects of cordycepin inhibit Aβ-induced apoptosis in hippocampal neurons. Neurotoxicology.

[29] Fan S., Zhang B., Luan P., Gu B., Wan Q., Huang X., Liao W., Liu J. (2015). PI3K/AKT/mTOR/p70S6K pathway is involved in Aβ25-35-Induced autophagy. BioMed Res. Int..

[30] Coronel R., Bernabeu-Zornoza A., Palmer C., Muniz-Moreno M., Zambrano A., Cano E., Liste I. (2018). Role of amyloid precursor protein (APP) and its derivatives in the biology and cell fate specification of neural stem cells. Mol. Neurobiol..

[31] Bernabeu-Zornoza A., Coronel R., Palmer C., Calero M., Martinez-Serrano A., Cano E., Zambrano A., Liste I. (2018). Aβ42 peptide promotes proliferation and gliogenesis in human neural stem cells. Mol. Neurobiol..

[32] Ahmad M.K., Abdollah N.A., Shafie N.H., Yusof N.M., Razak S.R.A. (2018). Dual-specificity phosphatase 6 (DUSP6): A review of its molecular characteristics and clinical relevance in cancer. Cancer Biol. Med..

[33] Wang Z., Chen Y., Li X., Sultana P., Yin M., Wang Z. (2018). Amyloid-β1-42 dynamically regulates the migration of neural stem/progenitor cells via MAPK-ERK pathway. Chem.-Biol. Interact..

[34] Harada T., Morooka T., Ogawa S., Nishida E. (2001). ERK induces p35, a neuron-specific activator of Cdk5, through induction of Egr1. Nat. Cell Biol..

[35] Giusti-Rodriguez P., Gao J., Graff J., Rei D., Soda T., Tsai L.H. (2011). Synaptic deficits are rescued in the p25/Cdk5 model of neurodegeneration by the reduction of β-secretase (BACE1). J. Neurosci..

[36] Kim Y., Rice A.E., Denu J.M. (2003). Intramolecular dephosphorylation of ERK by MKP3. Biochemistry.

[37] Zhang Z., Kobayashi S., Borczuk A.C., Leidner R.S., Laframboise T., Levine A.D., Halmos B. (2010). Dual specificity phosphatase 6 (DUSP6) is an ETS-regulated negative feedback mediator of oncogenic ERK signaling in lung cancer cells. Carcinogenesis.

[38] Allen Brain Atlas. http://mouse.brain-map.org/experiment/show/79632277.

[39] Jung S., Nah J., Han J., Choi S.G., Kim H., Park J., Pyo H.K., Jung Y.K. (2016). Dual-specificity phosphatase 26 (DUSP26) stimulates Aβ42 generation by promoting amyloid precursor protein axonal transport during hypoxia. J. Neurochem..

[40] Sanchez-Mut J.V., Aso E., Heyn H., Matsuda T., Bock C., Ferrer I., Esteller M. (2014). Promoter hypermethylation of the phosphatase DUSP22 mediates PKA-dependent TAU phosphorylation and CREB activation in Alzheimer’s disease. Hippocampus.

[41] Wang Y., Lin J., Chen Q.Z., Zhu N., Jiang D.Q., Li M.X., Wang Y. (2015). Overexpression of mitochondrial Hsp75 protects neural stem cells against microglia-derived soluble factor-induced neurotoxicity by regulating mitochondrial permeability transition pore opening in vitro. Int. J. Mol. Med..

[42] Nunes-Xavier C.E., Tarrega C., Cejudo-Marin R., Frijhoff J., Sandin A., Ostman A., Pulido R. (2010). Differential up-regulation of MAP kinase phosphatases MKP3/DUSP6 and DUSP5 by Ets2 and c-Jun converge in the control of the growth arrest versus proliferation response of MCF-7 breast cancer cells to phorbol ester. J. Biol. Chem..

[43] Wolvetang E.J., Wilson T.J., Sanij E., Busciglio J., Hatzistavrou T., Seth A., Hertzog P.J., Kola I. (2003). ETS2 overexpression in transgenic models and in Down syndrome predisposes to apoptosis via the p53 pathway. Hum. Mol. Genet..

[44] Um J.W., Kaufman A.C., Kostylev M., Heiss J.K., Stagi M., Takahashi H., Kerrisk M.E., Vortmeyer A., Wisniewski T., Koleske A.J. (2013). Metabotropic glutamate receptor 5 is a coreceptor for Alzheimer aβ oligomer bound to cellular prion protein. Neuron.

[45] Luan P., Zhou H.H., Zhang B., Liu A.M., Yang L.H., Weng X.L., Tao E.X., Liu J. (2012). Basic fibroblast growth factor protects C17.2 cells from radiation-induced injury through ERK1/2. CNS Neurosci. Ther..

[46] Oyarce K., Silva-Alvarez C., Ferrada L., Martinez F., Salazar K., Nualart F. (2018). SVCT2 is expressed by cerebellar precursor cells, which differentiate into neurons in response to ascorbic acid. Mol. Neurobiol..

[47] Fan D., Li J., Zheng B., Hua L., Zuo Z. (2016). Enriched environment attenuates surgery-induced impairment of learning, memory, and neurogenesis possibly by preserving BDNF expression. Mol. Neurobiol..

[48] Zhao Z.Y., Luan P., Huang S.X., Xiao S.H., Zhao J., Zhang B., Gu B.B., Pi R.B., Liu J. (2013). Edaravone protects HT22 neurons from H_2_O_2-_induced apoptosis by inhibiting the MAPK signaling pathway. CNS Neurosci. Ther..

[49] Fang W.L., Zhao D.Q., Wang F., Li M., Fan S.N., Liao W., Zheng Y.Q., Liao S.W., Xiao S.H., Luan P. (2017). Neurotropin (R) alleviates hippocampal neuron damage through a HIF-1/MAPK pathway. Cns Neurosci. Ther..

